# Virulence factors in environmental and clinical *Vibrio cholerae* from endemic areas in Kenya

**DOI:** 10.4102/ajlm.v3i1.41

**Published:** 2014-10-17

**Authors:** Racheal W. Kimani, Anne W.T. Muigai, Willie Sang, John N. Kiiru, Samuel Kariuki

**Affiliations:** 1 Institute of Tropical Medicine and Infectious Disease, Jomo Kenyatta University of Agriculture and Technology, Kenya; 2 Faculty of Science, Jomo Kenyatta University of Agriculture and Technology, Kenya; 3 Centre for Microbiology Research, Kenya Medical Research Institute, Kenya

## Abstract

**Background:**

Since 1971, Kenya has had repeated cholera outbreaks. However, the cause of seasonal epidemics of cholera is not fully understood and neither are the factors that drive epidemics, both in Kenya and globally.

**Objectives:**

The objectives of the study were to determine the environmental reservoirs of *V. cholerae* during an interepidemic period in Kenya and to characterise their virulence factors.

**Methods:**

One hundred (50 clinical, 50 environmental) samples were tested for *V. cholerae* isolates using both simplex and multiplex polymerase chain reaction.

**Results:**

Both sediments and algae from fishing and landing bays yielded isolates of *V. cholerae*. Clinical strains were characterised along with the environmental strains for comparison. All clinical strains harboured *ctxA, tcpA* (El Tor), *ompU, zot, ace, toxR, hylA* (El Tor) and *tcpI* genes. Prevalence for virulence genes in environmental strains was *hylA* (El Tor) (10%), *toxR* (24%), *zot* (22%), *ctxA* (12%), *tcpI* (8%), *hylA* (26%) and *tcpA* (12%).

**Conclusion:**

The study sites, including landing bays and beaches, contained environmental *V. cholerae*, suggesting that these may be reservoirs for frequent epidemics. Improved hygiene and fish-handling techniques will be important in reducing the persistence of reservoirs.

## Introduction

Since 1971, Kenya has suffered repeated cholera outbreaks. From 1974 to 1989, outbreaks were reported every year with an average case fatality rate of 3.6%.^1^ More cases have been reported in Kenya since 2005 and an outbreak in 2007 had a case fatality of up to 5.6%.^2^ In 2011, 60 cholera cases, including 10 laboratory-confirmed cases and one refugee death, were reported in the world’s largest refugee camp in Dadaab, Kenya.^3^

Significant advances have been made in understanding the molecular basis of *Vibrio cholerae* pathogenicity, including the identification of environmental reservoirs for the microorganism.^4^ It has also been shown that a 7th cholera pandemic spread from the Bay of Bengal in at least three independent but overlapping waves with a common ancestor in the 1950s and several transcontinental transmission events have been identified.^5^ The main reservoirs of *V. cholerae* are humans and aquatic sources such as brackish water and estuaries.^6^ Ahmed et al.^7^ showed that multiple genetic lineages of *V. cholerae* were simultaneously infecting persons in Kenya. This finding is consistent with the simultaneous emergence of multiple distinct genetic lineages of *V. cholerae* from endemic environmental reservoirs rather than recent introduction and spread by travelers. However, the cause of seasonal epidemics of cholera is not fully understood and neither are the factors that drive epidemics, both in Kenya and globally. The objectives of this study were to determine the environmental reservoirs of *V. cholera* during an interepidemic period in Kenya and to characterise their virulence factors.

## Research method and design

**FIGURE 1:**
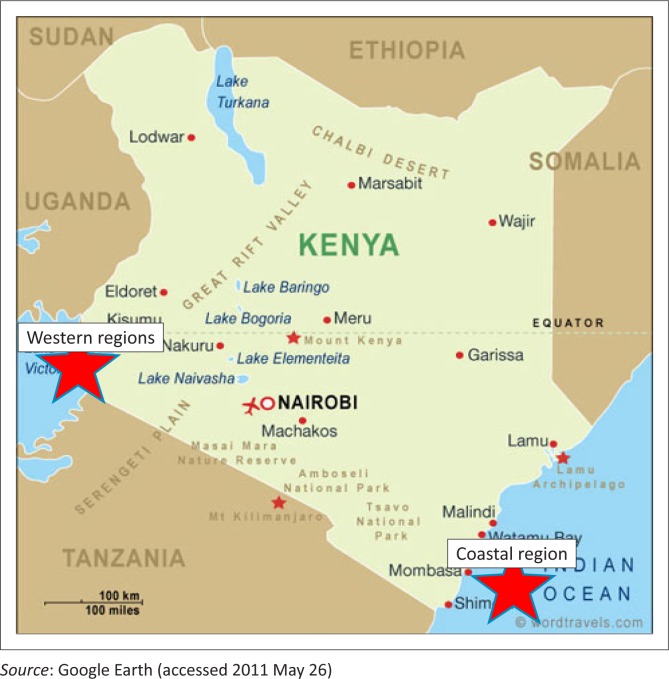
Coastal and western regions of Kenya where environmental samples were collected.

The study was conducted in the coastal and western regions of Kenya from February 2010 to November 2010. The coastal region sampling points were distributed in the cholera-endemic districts of Kwale, Malindi, Mombasa and Kilifi (Figure 1). At each sampling point, preference was given to watering points and where there was human activity, as recommended by local health officials. The western region sampling points included the districts of Kisumu, Siaya, Rachuonyo, Homa Bay, Nandi Hills, Nyando, Bondo, Vihiga and Busia. One sample of water, zooplankton, phytoplankton, sediments and/or floating vegetation was collected at each site as available, using the method described by Huq et al.^8^

Isolation began on the first day after collection of the environmental samples. One mL each of the zooplankton and phytoplankton homogenates was enriched in 10 mL (1X) alkaline peptone water. Ten mL of each plant homogenate and sediment sample were also enriched in 5 mL triple-strength alkaline peptone water. On the second day, subculturing was done from the previous day’s alkaline peptone water inoculates on plates of thiosulphate citrate bile salts (TCBS). On day three, the presence of yellow mucoid colonies was considered presumptive for *V. cholerae*.

Presumptive colonies were tested by measuring their reaction to an oxidase reagent (1% dimethyl-p-phenylene-diamine dihydrochloride), along with other biochemical tests (Voges Proskauer, Indole and carbohydrate fermentation). The environmental strains agglutinated with polyvalent O antisera and were sucrose fermenters. They formed large yellow mucoid colonies on the TCBS. Colonies were also gram stained to confirm the morphological characteristics of the isolates.

### Detection of virulence factors from environmental and clinical *V. cholerae*

Archived clinical isolates were sampled from the Kenya Medical Research Institute (KEMRI) laboratory. These isolates were mostly from outbreaks which occurred in the years 2009–2010. Systematic random sampling was carried out in order to choose clinical strains. From a list of whole population strain numbers, a random strain number was chosen as the start strain. Thereafter, every third strain from the whole population was sampled until a total of 50 samples were chosen, which is equal to the number of environmental strains.

### Amplification

Deoxyribonucleic acid (DNA) was isolated from freshly-cultured environmental and clinical *V. cholerae* isolates. The protocol was as follows: on the first day, bacterial isolates were grown at 37 ^°^C overnight in Mueller-Hinton plates. Cells were harvested and suspended in distilled water on the second day. The suspension was heated in a water bath at 95 ^°^C for five minutes to lyse the cells. This was then centrifuged at 14 000 x g for six minutes in order to remove the protein debris. A sample supernatant was then stored at -20 °C for further use. Polymerase chain reaction (PCR) was performed in a total volume of 32 µL containing 5 µL (5 ng/µL) of DNA template, 1.0 µL (0.1 mM) each of forward and reverse primers, Healthcare Mastermix (GE Healthcare, UK) and 25 µL nuclease-free water.

All strains were then screened for the presence of genes encoding virulence determinants in *V. cholerae* including cholera toxin (*ctxA*), zonula occludens toxin (*zot*), accessory cholera enterotoxin (*ace*), haemolysin (*hlyA*), and NAG-specific heat-stable toxin (*st*). Detection of the *tcpA* gene specific to the El Tor and Classical biotypes was performed using a common forward primer and biotype-specific reverse primers. Similarly, two forward primers were used for the detection of the biotype-specific haemolysin gene (*hylA*). PCR conditions and primers used for the detection of *tcpA, ompU, tcpI, toxR* and *hylA* genes were similar to those described previously by Rivera et al.^9^ (Table 1 and Table 2), whilst detection of the *ctxA* gene was done using primers and conditions described before by Fields et al.^10^ All PCR assays were performed using an automated Dynax thermal cycler. PCR products were analysed by electrophoresis on 1.5% agarose gels, stained with ethidium bromide, visualised under UV light and recorded with the aid of a gel-documentation system.

**TABLE 1:** Primers used in the study.

Primer	Sequence 5’-3’	Amplicon size in bp	Reference
*ctx*A-F	CGGGCAGATTCTAGACCTCCTG	564	Fields et al. 1992^10^
*ctx*A-R	CGATGATCTTGGAGCATTCCCAC		
*zot*-F	TCGCTTAACGATGGCGCGTTTT	947	Rivera et al. 2001^9^
*zot*-R	AACCCCGTTTCACTTCTACCCA		
*ace*-F	TAAGGATGTGCTTATGATGGACACCC	289	Shi et al. 1998^21^
*ace*-R	CGTGATGAATAAAGATACTCATATAGG		
*st*-F	GAGAAACCTATTCATTGC	216	Vicente et al. 1997^22^
*st*-R	GCAAGCTGGATTGCAAC		
*ompU*-F	ACGCTGACGGAATCAACCAAAG	869	Rivera et al. 2001^9^
*ompU*-R	GCGGAAGTTTGGCTTGAAGTAG		
*tcpI*-F	TAGCCTTAGTTCTCAGCAGGCA	862	Rivera et al. 2001^9^
*tcpI*-R	GGCAATAGTGTCGAGCTCGTTA		
*toxR*-F	CCTTCGATCCCCTAAGCAATAC	779	Rivera et al. 2001^9^
*toxR*-R	GGGTTAGCAACGATGCGTAAG		
*tcpA*-R (Classical)	TTACCAAATGCAACGCCGAATG	620	Rivera et al 2001^9^
*tcpA*-R (El Tor)	CGAAAGCACCTTCTTTCACACGTTG	453	Rivera et al 2001^9^
*tcpA*-F	CACGATAAGAAAACCGGTCAAGAG		
hylA-F (Classical)	GGCAAACAGCGAAACAAATACC	738	Rivera et al 2001^9^
hyl-F (El Tor)	GAGCCGGCATTCATCTGAAT	481	Rivera et al 2001^9^
*hylA-*R	CTCAGCGGGCTAATACGGTTTA		
SXT-F	ATGGCGTTATCAGTTAGCTGGC	1035	Bhanumathi et al. 2003^23^
SXT-R	GCGAAGATCATGCATAGACC		

*Source*: Publications as cited in Reference column

**TABLE 2:** Primers pooled for multiplex PCR analysis.

1st set of primers	2nd set of primers	3rd set of primers
*ompU*	*zot*	*ctxA*
*toxR*	*hylA* (Classical)	*tcpI*
*tcpA* (Classical)	*tcpA* (El Tor)	-
*hylA* (El Tor)	-	-

## Results

### Isolation of *V. cholerae* from environmental sources

The coastal region had a total of 23 positive isolates out of 207 collected environmental samples (11%) (Table 3). Kwale had the most positive isolates, accounting for 43% of positive isolates collected in this region, followed by Kilifi (35%) and Mombasa (22%). No samples collected in Malindi had positive isolates. The Western region had 27 positive isolates out of 199 collected samples (12%). Homa Bay had the most positive isolates, accounting for 30% of positive isolates collected in this region, followed by Kisumu, Siaya and Rachuonyo (15% each); Nandi Hills and Nyando (7% each) and Bondo, Vihiga and Busia (~4% each).

**TABLE 3:** Sources of *V. cholerae* samples collected in coastal and western Kenya by region.

Regions	Total number of samples collected	Positive isolates	Sample sources (Number of positive isolates/Total number collected)^1^							
			Mosses	Sediments	Floating water plants	Fish	Fish Offal	Water	Algae	Shrimp
**Coastal region**										
Kwale	104	10	-	1/36	4/24	2/7	-	0/38	3/9	-
Mombasa	41	5	-	0/11	0/2	0/7	-	3/13	2/8	-
Malindi	44	0	-	0/16	0/12	-	-	0/14	0/2	-
Kilifi	18	8	-	4/8	0/1	-	-	2/3	2/6	-
**Total**	**207**	**23**	**-**	**5**	**4**	**2**	**0**	**5**	**7**	**-**
**Western region**										
Kisumu	59	4	1/5	2/15	1/14	0/7	0/5	0/6	0/2	0/5
Siaya	22	4	1/3	0/4	0/4	1/7	-	2/4	-	-
Bondo	10	1	-	0/2	0/2	1/6	-	-	-	-
Vihiga	12	1	-	0/6	0/1	-	-	0/2	1/3	-
Nandi Hills	26	2	-	1/6	0/4	-	-	0/5	0/2	1/9
Busia	24	1	-	1/12	0/4	-	-	0/3	0/5	-
Homa Bay	25	8	1/6	3/8	1/2	0/1	1/3	-	2/5	-
Rachuonyo	18	4	-	1/6	0/2	0/4	-	2/3	1/3	-
Nyando	3	2	-	-	-	-	-	1/2	1/1	-
**Total**	**199**	**27**	**3**	**8**	**2**	**2**	**1**	**5**	**5**	**1**

### Detection of virulence genes from environmental and clinical *V. cholerae* isolates

**FIGURE 2:**
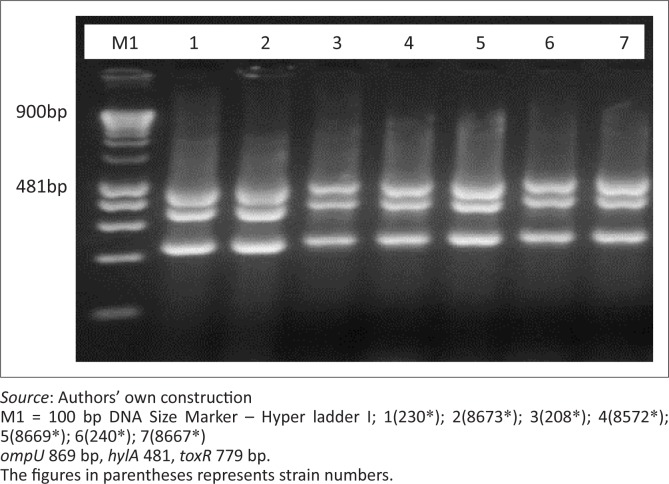
Example amplification of *ompU, toxR* and *hylA* genes in clinical *Vibrio cholera*.

All clinical strains (50 of 50) were PCR positive for the gene representing the El Tor fragment for haemolysin (*hylA*) (Figure 2), whilst only 10% (5 of 50) of the environmental strains were positive (Table 4). In addition, all clinical strains were PCR positive for the gene representing the Classical fragment for haemolysin as compared with 13 out of 50 (26%) of the environmental strains. For environmental *V. cholerae* strains, the results of the multiplex PCR yielded positive results for *tcpA* (El Tor) at 12% (6 of 50), *ctxA* at 8% (4 of 50), *zot* at 24% (12 of 50), *tcpI* at 8% (4 of 50), *toxR* at 22% (11 of 50) and o*mpU* at 0% (0 of 50) in the environmental strains. All clinical and environmental *V. cholerae* isolates (100%) were negative for the *st* gene.

**TABLE 4:** Results of Polymerase Chain Reaction.

Serial number of isolate	ctxΦ			VPI				*V. cholerae* haemolysin –lecithinase-lipase cluster			
				TCP Biogenesis			*toxR*				
	*ace*†	*ctxA*†	*zot*†	*tcpI*†	*tcpA* (Classical)†	*tcpA* (El Tor)†		*hylA* (Classical)†	*hylA* (El Tor)†	*st*†	*ompU*†
8677	+	+	+	+	-	+	+	+	+	-	+
8675	+	+	+	+	-	+	+	+	+	-	+
1419	+	+	+	+	-	+	+	+	+	-	+
8666	+	+	+	+	-	+	+	+	+	-	+
8674	+	+	+	+	-	+	+	+	+	-	+
247	+	+	+	+	-	+	+	+	+	-	+
229	+	+	+	+	-	+	+	+	+	-	+
241	+	+	+	+	-	+	+	+	+	-	+
231	+	+	+	+	-	+	+	+	+	-	+
8676	+	+	+	+	-	+	+	+	+	-	+
202	+	+	+	+	-	+	+	+	+	-	+
8679	+	+	+	+	-	+	+	+	+	-	+
1510	+	+	+	+	-	+	+	+	+	-	+
8678	+	+	+	+	-	+	+	+	+	-	+
50	+	+	+	+	-	+	+	+	+	-	+
49	+	+	+	+	-	+	+	+	+	-	+
8887	+	+	+	+	-	+	+	+	+	-	+
161	+	+	+	+	-	+	+	+	+	-	+
8888	+	+	+	+	-	+	+	+	+	-	+
8889	+	+	+	+	-	+	+	+	+	-	+
44	+	+	+	+	-	+	+	+	+	-	+
8884	+	+	+	+	-	+	+	+	+	-	+
Ruiru44	+	+	+	+	-	+	+	+	+	-	+
9487	+	+	+	+	-	+	+	+	+	-	+
1607	+	+	+	+	-	+	+	+	+	-	+
8880	+	+	+	+	-	+	+	+	+	-	+
VC003	+	+	+	+	-	+	+	+	+	-	+
46	+	+	+	+	-	+	+	+	+	-	+
8881	+	+	+	+	-	+	+	+	+	-	+
51	+	+	+	+	-	+	+	+	+	-	+
9503	+	+	+	+	-	+	+	+	+	-	+
VC133	+	+	+	+	-	+	+	+	+	-	+
VC134	+	+	+	+	-	+	+	+	+	-	+
64	+	+	+	+	-	+	+	+	+	-	+
168	+	+	+	+	-	+	+	+	+	-	+
238	+	+	+	+	-	+	+	+	+	-	+
8885	+	+	+	+	-	+	+	+	+	-	+
225	+	+	+	+	-	+	+	+	+	-	+
8886	+	+	+	+	-	+	+	+	+	-	+
8671	+	+	+	+	-	+	+	+	+	-	+
207	+	+	+	+	-	+	+	+	+	-	+
8667	+	+	+	+	-	+	+	+	+	-	+
230	+	+	+	+	-	+	+	+	+	-	+
8673	+	+	+	+	-	+	+	+	+	-	+
208	+	+	+	+	-	+	+	+	+	-	+
8572	+	+	+	+	-	+	+	+	+	-	+
8669	+	+	+	+	-	+	+	+	+	-	+
240	+	+	+	+	-	+	+	+	+	-	+
**Environmental isolates**											
1	-	-	-	-	-	+	-	+	-	-	-
2	-	-	-	-	-	+	+	-	-	-	-
3	-	+	-	+	-	+	+	-	-	-	-
4	-	-	-	+	-	-	-	-	-	-	-
6	-	-	-	-	-	+	-	-	-	-	-
7	-	-	-	-	-	+	-	-	-	-	-
10	-	+	-	-	-	-	-	-	-	-	-
11	-	-	-	-	-	+	-	-	-	-	-
14	+	-	+	-	-	-	+	+	-	-	-
18	-	+	-	-	-	-	-	-	-	-	-
19	-	-	+	-	-	-	-	+	-	-	-
22	-	+	-	+	-	-	-	-	-	-	-
20	-	-	-	-	-	-	-	-	-	-	-
23	-	-	-	-	-	-	-	-	-	-	-
27	-	-	-	-	-	-	-	-	-	-	-
31	-	-	-	-	-	-	-	-	-	-	-
32	-	-	-	-	-	-	+	-	-	-	-
34	-	-	-	-	-	-	-	-	-	-	-
35	-	-	-	-	-	-	+	+	-	-	-
40	-	-	-	-	-	-	-	-	-	-	-
41	-	-	+	-	-	-	-	+	-	-	-
43	-	-	+	-	-	-	-	-	-	-	-
42	-	-	-	-	-	-	-	+	-	-	-
58	-	-	+	-	+	-	+	+	+	-	-
55	-	-	-	-	+	-	-	+	-	-	-
36	-	-	+	-	-	-	-	-	-	-	-
37	-	-	+	-	-	-	+	-	+	-	-
44	-	-	-	-	-	-	-	-	-	-	-
56	-	-	-	-	-	-	-	+	-	-	-
46	-	-	+	-	-	-	+	+	-	-	-
25	-	-	+	-	-	-	+	+	-	-	-
48	-	-	+	-	-	-	+	-	-	-	-
50	-	-	-	-	-	-	+	-	-	-	-
29	-	-	-	-	-	-	-	-	-	-	-
47	-	-	-	-	-	-	-	-	-	-	-
54	-	-	-	-	-	-	-	-	+	-	-
51	-	-	-	-	-	+	-	-	+	-	-
38	-	-	-	-	-	-	-	-	-	-	-
26	-	-	-	-	-	-	-	-	-	-	-
56	-	-	-	-	-	-	-	-	-	-	-
57	-	-	-	-	-	-	-	+	-	-	-
16	-	-	-	-	-	-	-	-	-	-	-
8	-	-	-	-	-	-	-	-	+	-	-
15	-	-	-	-	-	-	-	-	-	-	-
21	-	-	-	-	-	-	-	-	-	-	-
52	-	-	-	-	-	-	-	-	-	-	-
53	-	-	-	-	-	-	-	-	-	-	-
33	-	-	+	+	-	-	-	-	-	-	-

*Source*: Authors’ own construction

Missing numbers for environmental isolates indicate samples that failed to grow or could not be used in assays after storage.

† Clinical Isolates.

+positive, - negative.

## Ethical considerations

Ethical approval was provided by the Kenya National Ethics review committee (approval number KEMRI/RES/7/3/1).

### Potential benefits and hazards

There were no potential benefits or hazards to human subjects as they were not involved in the study.

## Trustworthiness

### Reliability

Reliability is associated with accuracy, stability, consistency and reproducibility of the research. This was ensured by peer examination and open discussion with all participants.

### Validity

Validity entails ensuring that the datasets gathered or items used are pertinent or relevant to the research conducted. This was ensured by use of appropriate research design and methods.

## Discussion

### Isolation of environmental *V. cholerae* from Western and Coastal regions of Kenya

Knowledge regarding environmental reservoirs of *V. cholerae* is of critical epidemiological and public health importance. We identified environmental reservoirs of *V. cholera* in Kenya in a disease climate consisting of few reported cases. It is therefore likely that the *V. cholerae* existing in the environment amplify during the off-season to cause epidemics.

### Prevalence of virulence factors in environmental and clinical isolates of *V. cholerae*

In this study, the occurrence and distribution of selected virulence-associated genes in environmental and clinical strains of *V. cholerae* collected in Kenya were demonstrated.

Environmental studies of *V. cholerae* have been done with the expectation that the *V. cholerae* strains possessing the entire complement of virulence genes would be isolated. In this study, as in previous similar studies, this did not happen. This can be explained by the fact that virulence genes are dispersed amongst environmental strains of *V. cholerae* and may be ferried about in mobile genetic elements. These genotypes of environmental *V. cholerae* are said to act as reservoirs for the virulence factors and potential mixing and matching leads to the formation of new pathogenic strains.^11^ Two multiplex PCR assays revealed that all of the clinical strains were positive for *ctx, zot, tcpI*, and *ace*, as well as *hlyA* (both El Tor and Classical), *ompU* and the *toxR* gene, suggesting the presence of an intact core toxin region in all isolates. These genes are found together and represent the genome of filamentous bacteriophage CTXΦ. This implies that they possessed a CTXΦ and *tcp* pathogenicity island. The pilus colonisation factor TCP acts as a receptor for CTXΦ, which can infect non-toxigenic *V. cholerae*, leading to the emergence of new toxigenic strains of *V. cholerae*. The genes that encode the cholera toxin subunits *ctxA* and *ctxB* are localised to a CTX genetic element which is made up of a 4.6 Kbp central core region and a 2.4 Kbp repetitive sequence known as RS2.^12^ Classical strains of *V. cholerae* O1 contain two copies of the CTX element, one on each chromosome. Waldor and Mekalanos^13^ were able to show that CTX is a filamentous bacteriophage related to the M13 coliphage.^12^ Faruque, Alberts and Mekalanos^14^ reported that an environmental *V. cholerae* and two *V. mimicus* strains, all of which lacked TCP, were capable of being infected by CTXΦ.

The *V. cholerae* pathogenicity island (VPI) is 39.5 kb in size and contains genes associated with virulence (TCP-ACF cluster), the regulation of virulence (*toxT* and *tcpP/H*), the regulation of chemotaxis (*tcpI* or *acfB*) and mobility (*int* and *orfI*).^9^ The genes encoding TCP have been suggested to be part of a larger genetic element consisting of a cluster of genes. The *tcpA* El Tor gene was positive in all clinical *V. cholerae* isolates and in 12% of environmental isolates in the current study, and the *tcpA* Classical gene was negative in all isolates.

Harkey et al.^15^ suggested that regulators such as TcpI that act downstream of ToxR and ToxT may function to fine tune the expression of the TCP virulence determinant throughout the pathogenic cycle of *V. cholerae*. The TcpI encoding gene present in 8% of our environmental strains may thus have some other physiological functions as well.^16^

The outer membrane protein, OmpU, was reported to be a potential adherence factor for *V. cholerae*. In this study, the gene was present in all of the *V. cholerae* strains of clinical origin that were tested, and negative in *V. cholerae* strains isolated from the environment. The haemolysin gene has been employed to differentiate between the two biotypes of *V. cholera* O1. Two primer sets for the Classical and El Tor biotypes were used in this study. The *V. cholerae* strains of clinical origin from both biotypes were positive for the *hylA* gene. Environmental isolates were 26% positive for haemolysin of the Classical biotype and 10% for the El Tor biotype. This phenomenon of detecting both fragments encoding for *hylA* specific to both the Classical and the El Tor biotypes in one isolate was observed in a similar study.^17^ NAG-ST in *V. cholera* O1 strains that is closely related to the heat-stable toxins produced by enterotoxigenic *E. coli* and other enteric pathogens was defined.^18^ The heat-stable enterotoxin production by the *st* genes was tested and none of the *V. cholerae* strains (of either environmental or clinical origin) were positive.

The regulation and expression of genes for growth and survival depend on the regulon ToxR, which is regulated by a cascade mechanism involving three known components: ToxR, ToxS and ToxT. ToxR, a 32-kDa transmembrane protein, is the master regulator and its expression is dependent upon environmental growth conditions (incubation temperature, pH, osmolarity, bile salts, oxygen tension, hydrostatic pressure and amino acid composition of the medium.^19^ The *toxR* gene encodes a transcriptional activator controlling CT gene expression (*ctxA*), TCP biogenesis (*tcpA*), outer membrane protein expression (*ompU*), and at least 17 distinct genes in the O139 and O1 strains.^20^ In this study, the presence of the *toxR* gene was verified in all *V. cholerae* isolates of clinical origin and in 24% of the environmental isolates.

## Conclusion

The implications of finding *V. cholerae* in environmental sources with virulence genes also found in clinical isolates are significant. The possibility is that this may lead to an epidemic strain arising through gene transfer that links genes that facilitate transmission in human populations, whilst those that foster persistence are proliferating in nearby environmental niches. Gene interactions are therefore important to understanding the epidemiology and persistence of virulence factors and subsequent epidemic outbreaks. Proper hygiene and fish-handling techniques should be ensured to cut the transmission from environmental sources to humans.
